# Shoot tip culture: a step towards 13C metabolite flux analysis of sink leaf metabolism

**DOI:** 10.1186/s13007-019-0434-8

**Published:** 2019-05-20

**Authors:** Somnath Koley, Manish L. Raorane, Björn H. Junker

**Affiliations:** Institute of Pharmacy, Martin Luther University, Hoher Weg 8, Halle (Saale), Germany

**Keywords:** Sink leaves, *Mentha piperita*, Shoot culture, Secondary metabolism, Metabolic flux analysis

## Abstract

**Background:**

Better understanding of the physiological and metabolic status of plants can only be obtained when metabolic fluxes are accurately assessed in a growing plant. Steady state ^13^C-MFA has been established as a routine method for analysis of fluxes in plant primary metabolism. However, the experimental system needs to be improved for continuous carbon enrichment from labelled sugars into metabolites for longer periods until complex secondary metabolism reaches steady state.

**Results:**

We developed an in vitro plant culture strategy by using peppermint as a model plant with minimizing unlabelled carbon fixation where growing shoot tip was strongly dependent on labelled glucose for their carbon necessity. We optimized the light condition and detected the satisfactory phenotypical growth under the lower light intensity. Total volatile terpenes were also highest at the same light. Analysis of label incorporation into pulegone monoterpene after continuous U-^13^C_6_ glucose feeding revealed nearly 100% ^13^C, even at 15 days after first leaf visibility (DALV). Label enrichment was gradually scrambled with increasing light intensity and leaf age. This study was validated by showing similar levels of label enrichment in proteinogenic amino acids. The efficiency of this method was also verified in oregano.

**Conclusions:**

Our shoot tip culture depicted a method in achieving long term, stable and a high percentage of label accumulation in secondary metabolites within a fully functional growing plant system. It recommends the potential application for the investigations of various facets of plant metabolism by steady state ^13^C-MFA. The system also provides a greater potential to study sink leaf metabolism. Overall, we propose a system to accurately describe complex metabolic phenotypes in a growing plant.

**Electronic supplementary material:**

The online version of this article (10.1186/s13007-019-0434-8) contains supplementary material, which is available to authorized users.

## Background

The ever-increasing demand for food [1, 2] coupled with increasing dependence on plants as a primary source of medicines (supplying 50% of modern drugs) [3, 4] has heightened the need to optimise plant metabolism. Despite the discovery of new enzymes and their regulatory controls, pathways specific to cell and/or organelle, developmental stage and biotic/abiotic stress response are still very ambiguous. Also, the intracellular metabolic fluxes are adapted as per the demands and the physiological state of the cell [5], so information about metabolic fluxes cannot be generalized at any spatial or temporal level. Thus, additional information on metabolic fluxes is even more imperative than the metabolites themselves towards determining an authentic metabolic phenotype [6, 7].

Typically, positionally labelled ^13^C substrates have been key players towards the development of the ^13^C metabolic flux analysis (MFA) approach in plants, in which the metabolic fate of the substrate further allows us to infer more accurately the relative fluxes through different metabolic networks [8, 9]. Recently, there has been substantial progress in ^13^C tracer studies, however, usually isolated plant tissues with homogenous cell populations and long metabolic steady state have been extensively investigated using this technique. Central metabolic fluxes were characterised in cultured oilseeds, developing embryos of Arabidopsis and Brassica genotypes to imply on potential metabolic engineering targets towards improved yield and better seed composition [10–13]. Likewise, differentiated systems such as plant hairy root cultures of *Catharanthus roseus* and tobacco were also studied to understand the metabolic impact of genetic and environmental changes [14, 15].

One of the major challenges for steady state ^13^C-MFA in plants is the accomplishment of the steady state at both the isotopic and metabolic level. The time factor required to achieve both steady states has to be short to avoid significant metabolic shifts, which might further lead to a misconception of the ^13^C-MFA study [16]. This is the reason why the general focus of steady state MFA mainly revolves around central core metabolism where a steady state for metabolites can be established within seconds to several minutes or hours [17–19]. However, studies involving secondary metabolism networks require much longer experimental periods to establish the metabolic steady state. In such cases, transient isotope labelling approaches were used to estimate accurate metabolic fluxes as the conventional steady state approach lacks conviction [20, 21]. Another challenge for steady state ^13^C-MFA is the application to plant tissues, which is limited by a high heterogeneity of the involved cell types and, furthermore, a number of subcellular compartments. Studies on developing maize seeds have used tissues that have been cultured separately or in pairwise combination to study central metabolic fluxes [10, 22]. However, in such heterogeneous systems, the actual metabolism is difficult to determine. It becomes even more critical when studying growing plant system using isotopic sugars, where photosynthesis has the capacity to limit the label enrichment of the metabolites. Feeding the system with ^13^CO_2_ substrate instead of the sugars allows quantifying photoautotrophic metabolic fluxes, however only at the transient state of isotope incorporation, as the steady state will be achieved independent of the flux distribution [23, 24]. Thus, the conventional ^13^C-MFA study is capable of quantifying heterotrophic and mixotrophic metabolic fluxes as shown by several studies within the literature. Hence to improve the practicability of the steady state ^13^C-MFA on growing plants, the above-mentioned constraints are needed to be addressed.

When high label enrichments should be achieved in mixotrophic tissues such as sink leaves, it is crucial to decrease photosynthetic carbon fixation without affecting other physiological processes. In order to maintain sink tissue as a net importer for the longer time, light irradiation can be reduced. Light correlates positively with plant growth, however negatively with label enrichment from ^13^C sugar tracers by regulating photosynthetic rate [25]. A recent study showed the ^13^C enrichment in Arabidopsis sink leaves for short dark periods (4 h), where label enrichment from U-^13^C_6_ sucrose feeding was up to 34.8% in different amino acids [26]. In that study, a conventional shoot culture method with the presence of source leaves as explant were explored for central metabolism. However, label amounts can even be further increased by minimizing the initial biomass prior to the experiment.

Here, we introduce a system to increase the potential for long-term isotopic steady state labelling to probe intact plant systems for a better understanding of secondary metabolism. The confounding effect of growing plant system to perform steady state MFA has been addressed here using a shoot-tip culture system. The method was established in growing peppermint (*Mentha piperita*), a member of the *Lamiaceae* family. Peppermint has been a model system for studying monoterpene biosynthesis due to its ability to produce extensive amounts of commercially important essential oil with antimicrobial, antiviral, antioxidant and antitumor actions [27, 28]. We also extended our method to oregano (*Origanum vulgare*), another member of the tribe *Mentheae* with high essential oil content, to further ascertain our strategy. The procedure is economically feasible, robust and easy to use and represents high labelling percentage for longer periods. Besides, an interesting strategy was devised to alter light intensity, which further inhibited the rate of light-dependent reactions and thus obtain a very high labelling efficiency in growing plant system. The system was also validated for primary metabolism with maximum 6% deviation of label enrichment between proteinogenic amino acids and secondary metabolites, even after a leaf growth period of 15 days. Thereby, we lay the foundations to trace carbon isotopes for studying sink leaf metabolism and to further increase the accuracy of estimations of metabolic fluxes within growing plants for longer durations.

## Results

We focused the development of our method on peppermint, a model plant for terpenoid biosynthesis. In our study, we first established a culture system using peppermint shoot tips. Stable isotope labelling experiments were performed on such a shoot tip culture system fed with exogenous labelled glucose to synthesize labelled monoterpenes. Monoterpenes are C_10_ compounds having (M + 0) to (M + 10) mass isotopomers, for example, the molecular weight of (M + 0) to (M + 10) mass isotopomer of pulegone is 152.23–162.23 Da. On using the source of entirely unlabelled glucose, theoretically, the lowest mass isotopomer (M + 0) depicts the highest accumulation, while in case of uniformly labelled glucose source, the highest mass isotopomer (M + 10) exhibits the highest accumulation. We shall then explicitly describe various approaches that were employed towards achieving and maintaining high ^13^C label enrichment within our new system and finally discuss the application of our method in studying ^13^C-MFA via sink leaf metabolism.

### Establishment of the ^13^C enriched shoot tip culture

**Fig. 1:**
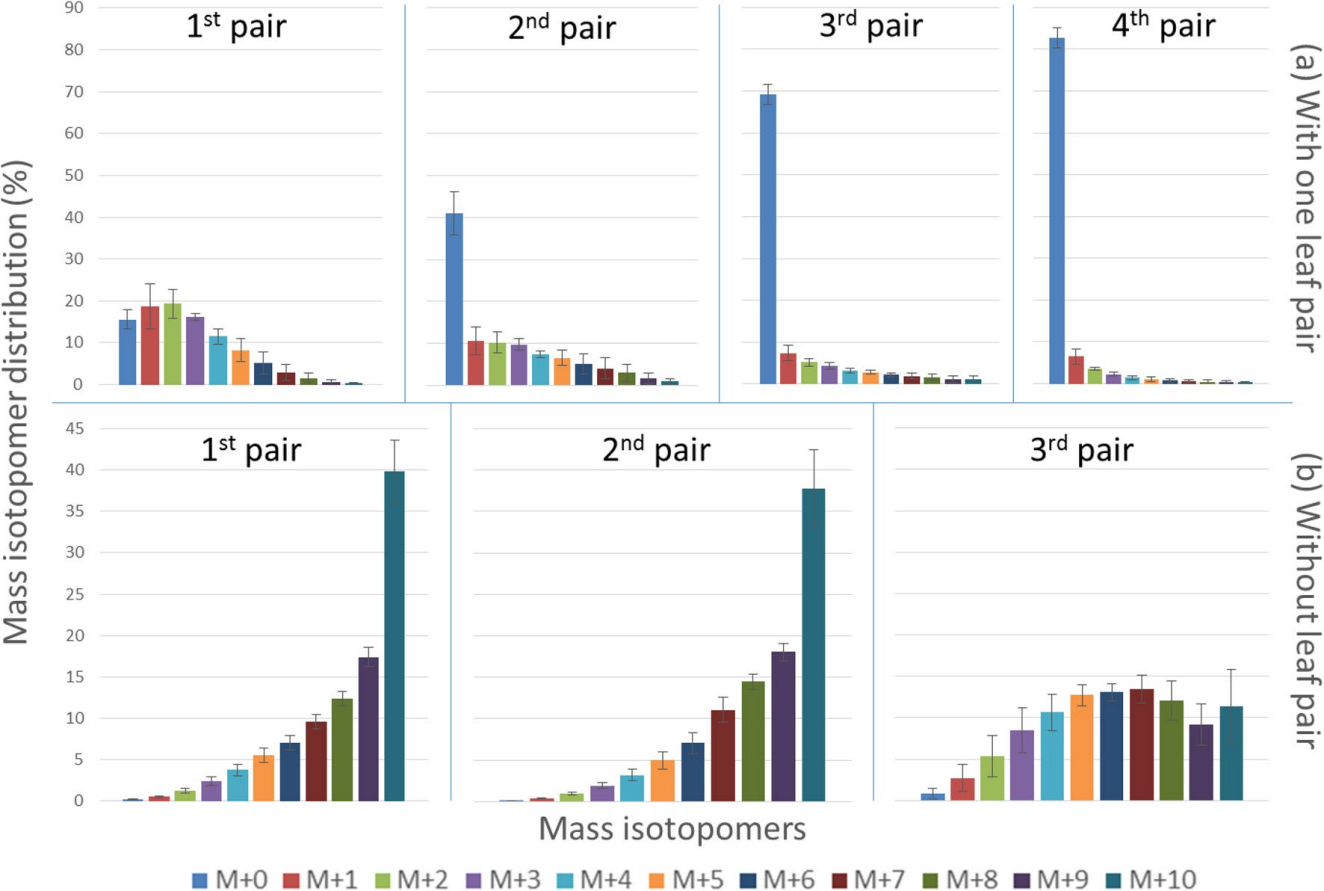
Mass isotopomer distribution (%) of pulegone in each leaf pair at 30 μmol m^−2^ s^−1^ light intensity. **a** Shoot tip culture initiated with one developing leaf pair and at after 15 days of culture total four leaf pairs (older than 6 days) were observed. **b** Shoot tip culture started without leaves and at 15 DALV total three leaf pairs (older than 6 days) were observed (mean ± standard error, n = 5)

**Fig. 2:**
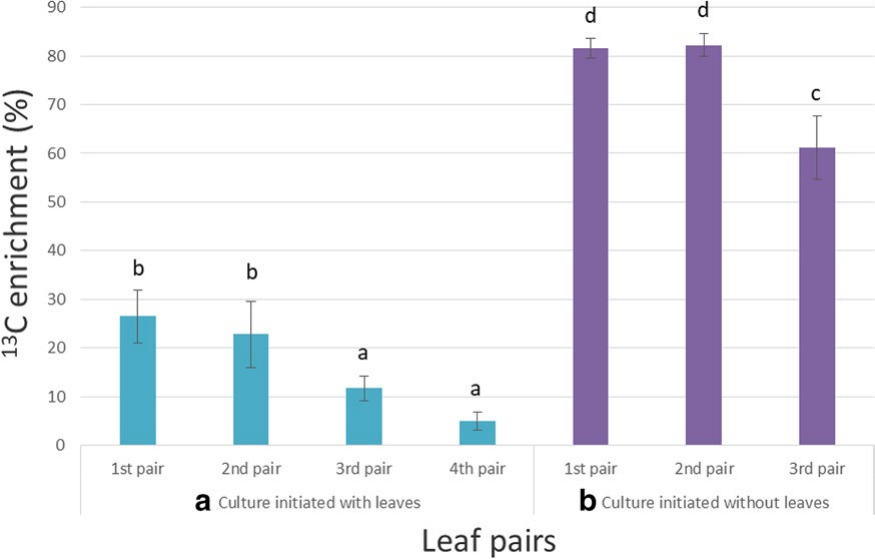
Average label enrichment (%) into pulegone of each leaf pair at 30 μmol m^−2^ s^−1^ light intensity. **a** Shoot tip culture initiated with one developing leaf pair and after 15 days of culture total four leaf pairs (older than 6 days) were observed. **b** Shoot tip culture started without leaves and at 15 DALV total three leaf pairs (older than 6 days) were observed. For statistics, data were evaluated by one-way Anova, followed by Tukey HSD test and statistical difference (p < 0.05) was indicated by different letters (a to d) (mean ± standard error, n = 5)

The shoot tip culture studies were performed in liquid medium as compared to solid agar medium, because of the convenience of maintaining the required glucose concentration over time. Each explant was cultivated in 3.5–4.0 ml liquid medium. In the basal Murashige and Skoog (MS) medium, glucose was used as a sole carbon source. Growth hormones and antibiotics were not added to the culture basal medium to minimize the dilution of ^13^C by unlabelled ^12^C in the culture system. The critical part to establish a predominantly labelled biological system was to minimize the primary unlabelled carbon source. Two strategies were devised in order to find the optimal starting material as an explant for the shoot tip culture system. Firstly, the shoot tip culture study (Fig. 1a) was initiated with one existing leaf pair and monitored for mass isotopomer distributions (MIDs) after 15 days of the cultivation at 30 µmol m^−2^ s^−1^ light intensity. In spite of feeding shoot tip with U-^13^C_6_ glucose, the (M + 10) distribution of the monoterpene pulegone was the lowest in each pair of leaves, whereas (M + 0) isotopomer was the highest in three new pairs of leaves (2nd to 4th pair). This indicated that label incorporation was insufficient and diluted over time with newer leaf pairs. As a consequence, ^13^C enrichment (Fig. 2a) was only 27% in the first leaf pair and gradually decreased in newer leaf pairs. However, higher label incorporation is desirable for better interpretation of ^13^C-MFA. Therefore, a second strategy was established wherein the existing leaf pair were excised and the explant was raised in similar conditions as described previously. The removal of existing leaves meant that the unlabelled source of carbon was reduced from the explant. After 15 DALV (M + 10) isotopomer was the highest in a first and second leaf pair (Fig. 1b). Labelled carbon was more diluted in the newest (3rd) pair of leaves. Similar kind of comparisons was also observed in other monoterpenes (Additional file 1: Figure S1 and S2). However, it was very evident that ^13^C was highly enriched (61–82%) in the second strategy compared to the first (5–27%) (Figs. 1b, 2b). Thus, we concluded that the shoot tip culture method without previously existing leaves was more suitable for ^13^C-MFA and was used for further analysis within the scope of this paper.

### Determination of the optimal light conditions through phenotypical characterisation

Light has indirect influence for unlabelled CO_2_ fixation from ambient air to plant. Hence, we studied the effects of different light intensities (5, 10, 20 and 30 µmol m^−2^ s^−1^) on ^13^C enrichment to increase the label incorporation even beyond the maximum observed from our shoot tip culture method establishment. Leaves were harvested at either 15 or 20 DALV, since monoterpene biosynthesis and total monoterpene content was noted to be pronounced at this respective leaf age [29]. The highest glucose uptake was 55 mg per shoot tip explant under studied light intensities for 15 DALV (Additional file 1: Table S1). Hence, the basal medium (3.5–4.0 ml) was supplemented with 2% w/v (70–80 mg) glucose, which was more than the required amount. We characterised our explants morphologically and physiologically to gain a deeper understanding of their growth status under different light intensities. Two morphological traits (Table 1), such as the total leaf weight and the number of leaves were determined. The lowest selected light intensity for the culture was 5 µmol m^−2^ s^−1^. Explants grown at lower than 5 µmol m^−2^ s^−1^ light intensity showed no visible leaf emergence. Leaf pairs, which were visible for more than 3 days, were counted. After 15 DALV, the total number of leaf pairs (2 pairs) remained the same for 5-20 µmol m^−2^ s^−1^ light intensity (Table 1, Additional file 1: Figure S3). However, the explant grown at 30 µmol m^−2^ s^−1^ light intensity, showed the development of an extra pair of leaves (3rd pair). Total leaf weight was also observed to increase with increasing light intensity and was significantly highest under 30 µmol m^−2^ s^−1^ light.

**Table 1:** Phenotypic traits of peppermint shoot tip culture for 15 DALV under different light intensities

Light intensities	Total leaf weight (mg)	Total number of leaves
5 µmol m^−2^ s^−1^	41.92 ± 3.55^a^	2 pairs
10 µmol m^−2^ s^−1^	43.44 ± 6.76^a^	2 pairs
20 µmol m^−2^ s^−1^	59.36 ± 3.05^b^	2 pairs
30 µmol m^−2^ s^−1^	90.89 ± 4.60^c^	3 pairs

For statistics, data were evaluated by one-way Anova, followed by Tukey HSD test and statistical difference (p < 0.05) was indicated by different letters (mean ± standard error, n = 5). Zero standard error of leaf number was calculated, as all observations of the same treatment were identical

In order to better understand the physiological status of these explants under different light intensities, amounts of chlorophyll a, b and carotenoids were quantified for both the shoot tip cultures as well as normal soil-grown peppermint plants in growth rooms. As presented in Table 2, chlorophyll a and b was notably higher at 30 µmol m^−2^ s^−1^ than at 5–10 µmol m^−2^ s^−1^ light. The normal plant also showed similar chlorophyll a and b contents to these shoot tip explants (Additional file 1: Table S2). The chlorophyll a to b ratio was also computed to get a better understanding of the adaptive response of the light-harvesting complex. We also observed significantly higher chlorophyll a to b ratio in the lower two light intensities compared to the maximum two cultured light conditions. Carotenoid contents were also unaffected at different light intensities.

**Table 2:** Production of photosynthetic pigments (mg g^−1^ leaf fresh weight) in leaves from 15 DALV

Light intensities	Chlorophyll (mg g^−1^)			Chl (a/b)	Carotenoids (mg g^−1^)
	Chl a	Chl b	Total (a + b)		
5 µmol/m^2^/s	1.11 ± 0.09^a^	0.66 ± 0.02^a^	1.77 ± 0.11^a^	1.68^a^	0.23 ± 0.02^a^
10 µmol/m^2^/s	1.15 ± 0.04^a^	0.76 ± 0.08^a^	1.91 ± 0.11^a^	1.52^a^	0.20 ± 0.01^a^
20 µmol/m^2^/s	1.27 ± 0.05^ab^	1.12 ± 0.04^b^	2.38 ± 0.04^b^	1.13^b^	0.20 ± 0.02^a^
30 µmol/m^2^/s	1.46 ± 0.11^b^	1.20 ± 0.07^b^	2.66 ± 0.09^c^	1.21^b^	0.22 ± 0.05^a^

Shoot tip culture reared under different light intensities (mean ± standard error, n = 5). For statistics, data were evaluated by one-way Anova, followed by Tukey HSD test and statistical difference (p < 0.05) was indicated by different letters

On the basis of this limited morpho-physiological characterisation, it can be deduced that the best development of plant was detected under higher light intensity, however, lower light intensity did not show any adverse effects on plant growth and its physiological status.

### Establishing optimal light conditions for the highest and stable ^13^C incorporation

**Fig. 3:**
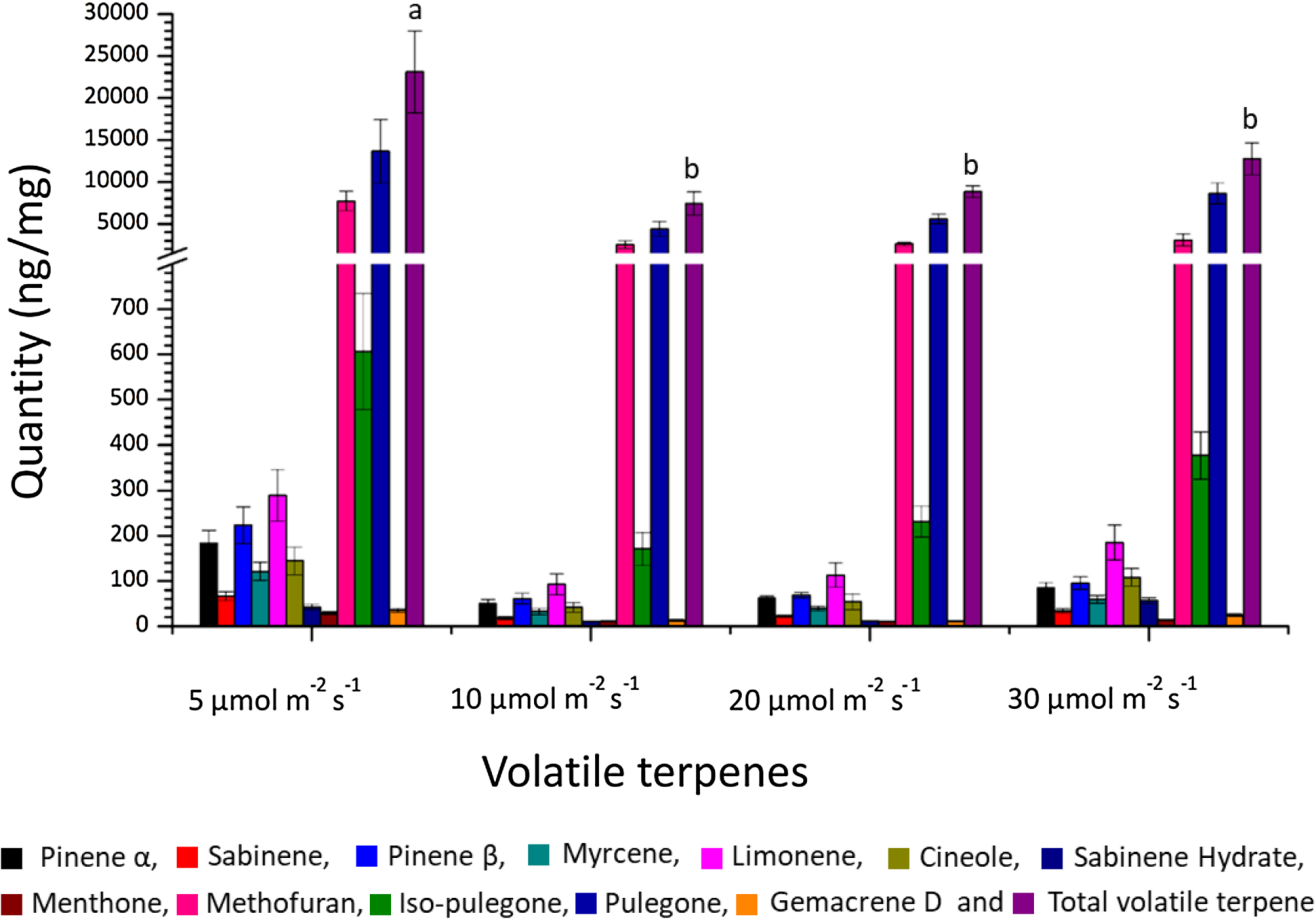
Comparison of volatile terpenes (ng/mg leaf fresh weight) produced under four different light intensities. 11 monoterpenes, 1 sesquiterpene (germacrene D) and total of those volatiles was found in 15 DALV old leaves. For statistics, total terpene amounts were evaluated by one-way Anova, followed by Tukey HSD test and statistical difference (p < 0.05) was indicated by different letters (a to b) (mean ± standard error, n = 5)

Once the establishment of optimal light conditions for satisfactory growth of explants in the shoot tip culture system was achieved, it was now imperative to optimise the conditions for higher and stable ^13^C incorporation in the plant terpenoids, which were the main focus of our study. Previously it was shown that volatile terpenes cannot be detected in peppermint leaves younger than 7 days [29], hence the leaves older than 6 days were further analysed for the terpene content. Qualitative and quantitative analysis was accomplished for eleven monoterpenes and one sesquiterpene from these shoot tip cultures under different light intensities (Fig. 3). Pulegone was the most abundant monoterpene in the leaves followed by menthofuran and isopulegone. The relative amounts of other monoterpenes and sesquiterpene were very limited. Interestingly, most of the monoterpenes (except pulegone, cineole, sabinene hydrate and gemacrene D) were apparently produced in higher amounts under the lowest light intensity. The maximum accumulation of the total volatile terpenoids per unit of leaf weight was also observed under 5 µmol m^−2^ s^−1^ light intensity.

**Fig. 4:**
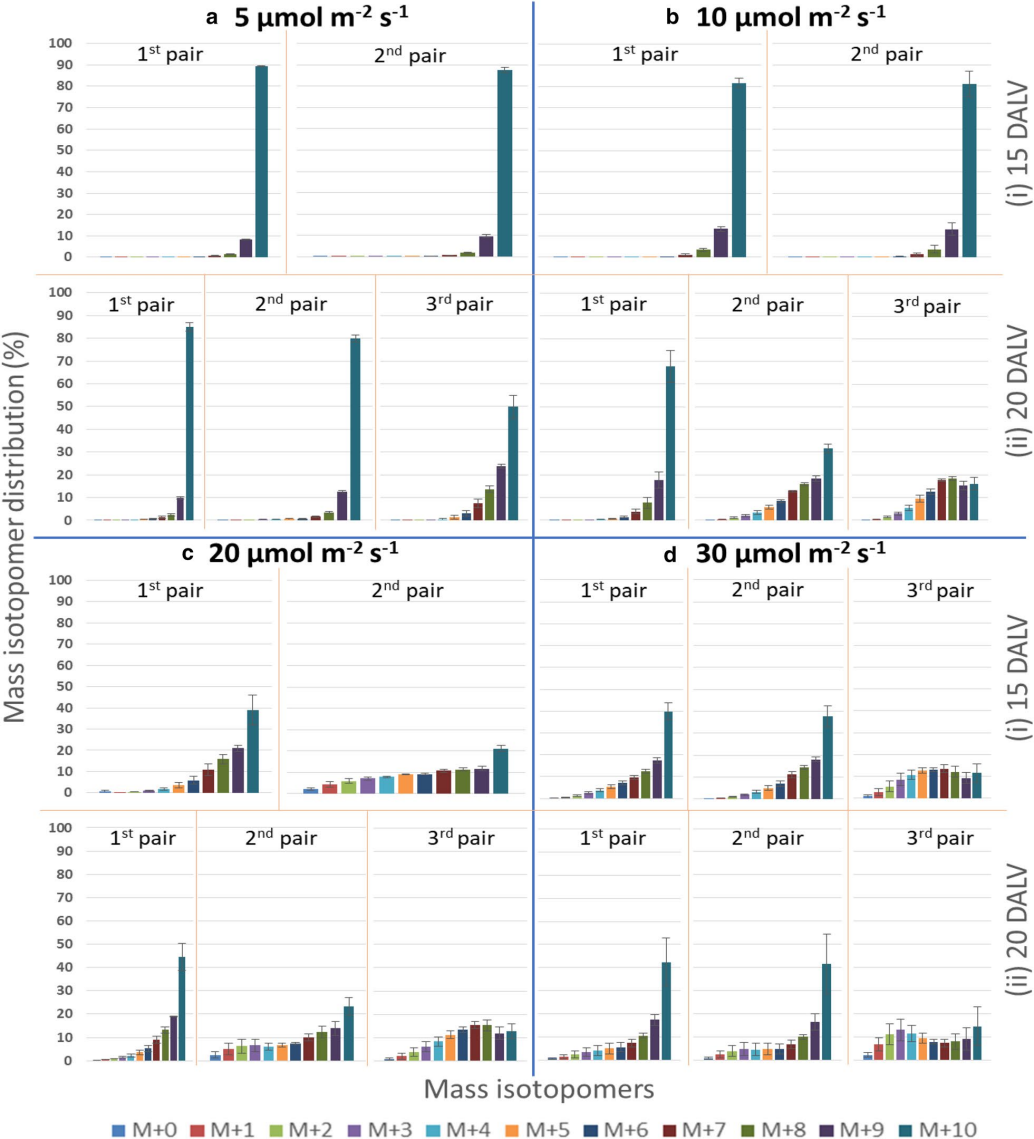
Mass isotopomer distribution (%) of pulegone in different leaf pair found under different light intensities. **a** shoot culture at 5 μmol m^−2^ s^−1^ light intensity, **b** shoot culture at 10 μmol m^−2^ s^−1^ light intensity, **c** shoot culture at 20 μmol m^−2^ s^−1^ light intensity and **d** shoot culture at 30 μmol m^−2^ s^−1^ light intensity. Measurement was taken on (i) 15 DALV and (ii) 20 DALV for each light intensity (mean ± standard error, n = 5)

Higher amounts of pulegone, allowed it to be selected as a prime candidate for further mass isotopomer distribution analysis. As a control, unlabelled glucose was used as a sole carbon source and the label distribution in (M + 0) isotopomer after natural abundance correction was accounted to be 99.74% (Additional file 1: Figure S4). Uniformly labelled glucose (U-^13^C_6_) replaced unlabelled glucose into the shoot tip culture medium to study the label incorporation patterns through the mass isotopomer distributions within pulegone. The highest accumulation of the (M + 10) isotopomer of pulegone in all pairs of leaves was observed under 5 µmol m^−2^ s^−1^ light condition, irrespective of their age (Fig. 4a). Such a desirable pattern of isotopomer distribution was also noticed in explants grown under 10 µmol m^−2^ s^−1^ light until 15 DALV (Fig. 4b). The shoots exposed to 20 and 30 µmol m^−2^ s^−1^ light intensities showed varied dilution of the labelled carbon within several mass isotopomers of pulegone (Fig. 4c, d).

While exploring the leaves of different age, it was apparent that the labelling percentage was decreased with the increasing age of shoot culture. For instance, the contribution of (M + 10) isotopomer of the second pair of leaves at 5 µmol m^−2^ s^−1^ was slightly declined from 89 to 80% with increasing age from 15 DALV to 20 DALV (Fig. 4a). Similarly, this dilution of the label was even more prominent under 10 µmol m^−2^ s^−1^ light (Fig. 4b). The amount of decreasing percentage in (M + 10) isotopomer was 13% and 50% in the first and second pair of leaves respectively (Fig. 4b) with advancing leaf age. No significant effects were observed within the (M + 10) isotopomer in the specific leaf pair from 15 DALV to 20 DALV (Fig. 4c, d) under higher two light conditions.

Comparison between different leaf pairs revealed that the newest leaf pair (3rd pair) always had the lowest abundance of (M + 10) isotopomer. Among pulegone of different leaf pairs found at 5 µmol m^−2^ s^−1^ light after 20 DALV, accumulation of highest isotopomer (M + 10) was markedly lower in third leaf pair (50%) (Fig. 4a). Moreover, this accumulation was continuously attenuated with each newer leaf pair of 20 DALV aged plant under 10 and 20 µmol m^−2^ s^−1^ light intensity (Fig. 4b, c). Although the first and second leaf pair from highest tested light intensity had significantly indifferent (M + 10) distribution, however, the third leaf pair had the least accumulation (Fig. 4d). Thus, the accumulation of label showed a significant difference between the leaf pairs for the plants exposed to different light intensities. The only exception to this comparison at a specific age was 15 DALV aged plants exposed to 5 and 10 µmol m^−2^ s^−1^ light intensity (Fig. 4a, b).

**Fig. 5:**
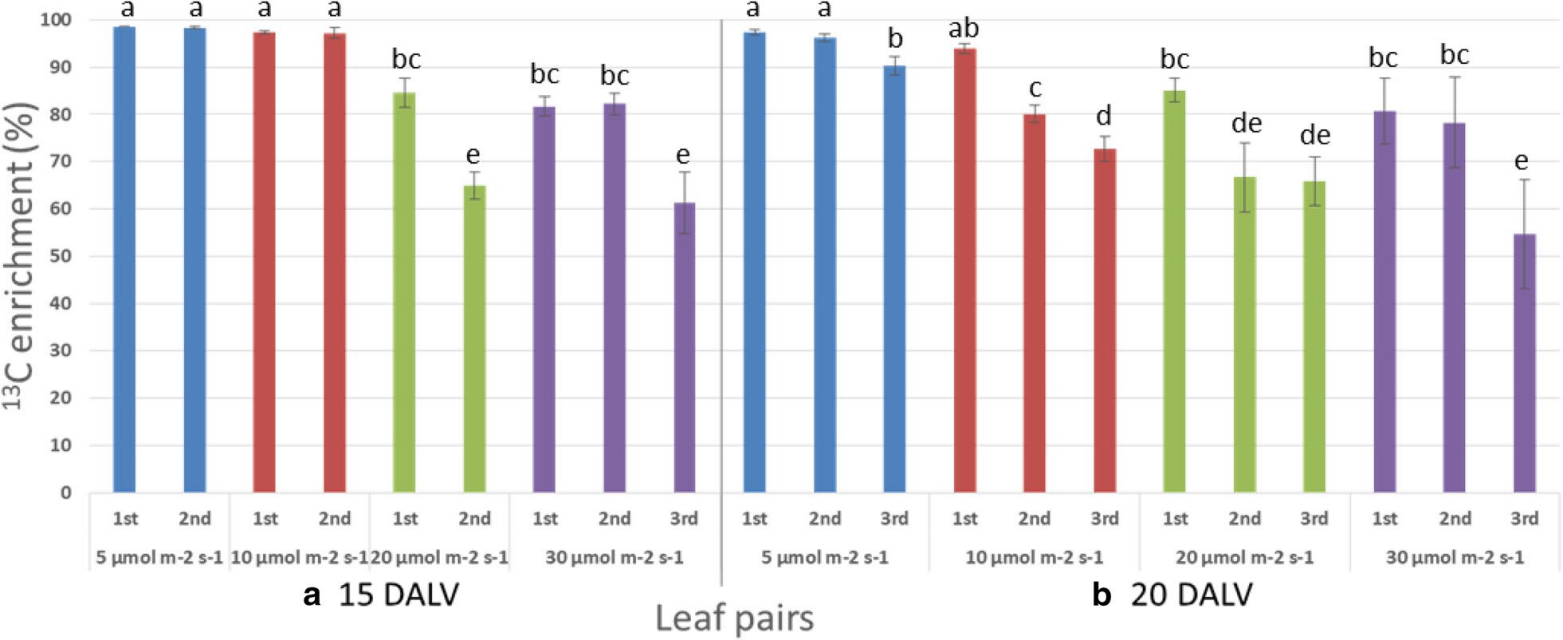
Average label enrichment (%) into pulegone in different leaf pair found under different light intensities. **a** at 15 DALV and **b** at 20 DALV. For statistics, data were evaluated by oneway Anova, followed by Tukey HSD test and statistical difference (p < 0.05) was indicated by different letters (a to e) (mean ± standard error, n = 5)

**Fig. 6:**
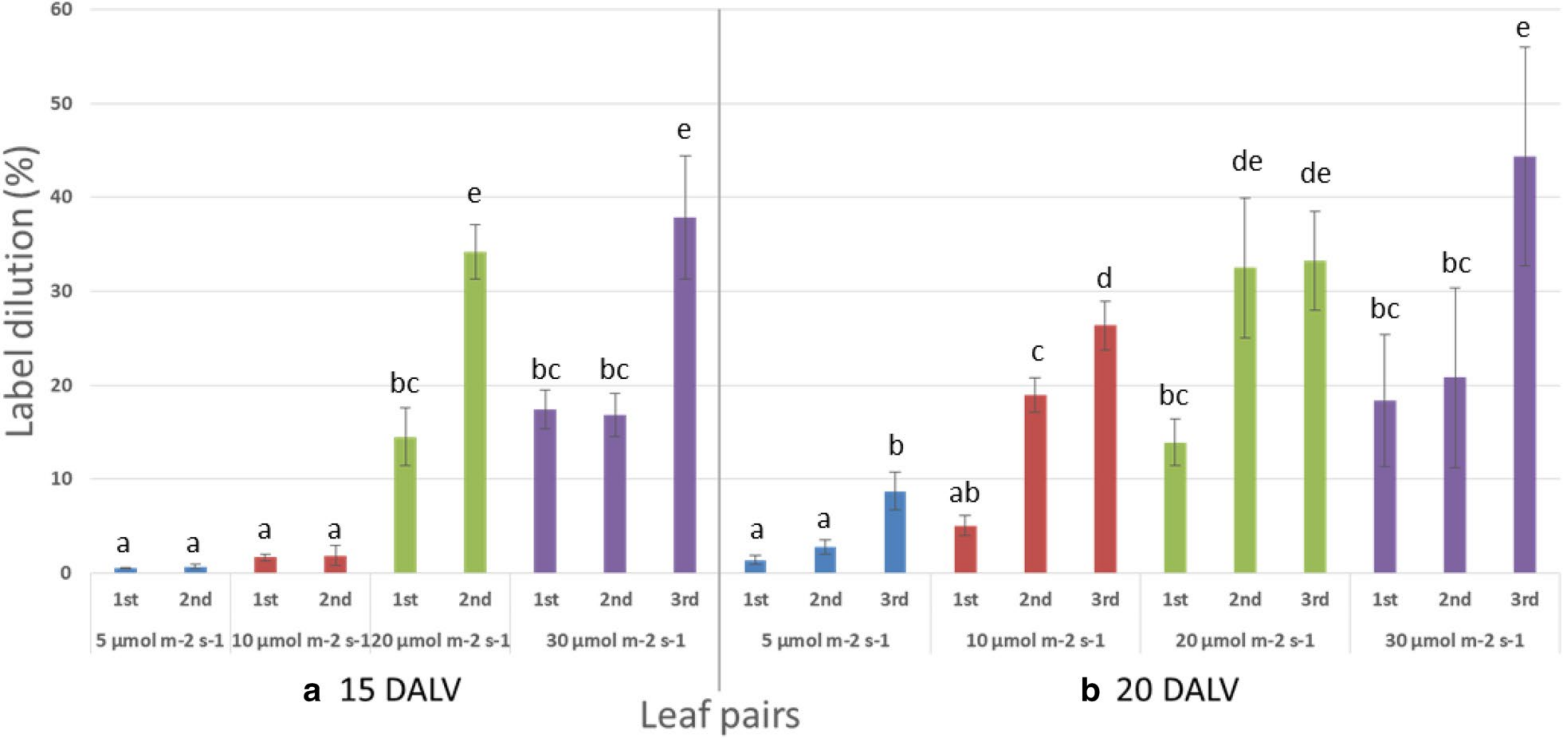
Average label dilution (%) into pulegone in different leaf pair found under different light intensities. **a** at 15 DALV and **b** at 20 DALV. For statistics, data were evaluated by one-way Anova, followed by Tukey HSD test and statistical difference (p < 0.05) was indicated by different letters (a to e) (mean ± standard error, n = 5)

^13^C enrichment and label dilution were calculated for more accurate and precise measurement of the label incorporation into pulegone. Correction of 1% ^12^C impurity in U-^13^C_6_ glucose was considered to determine the dilution of labelling appropriately. Measured dilutions were derived from either unlabelled carbon of initial explant or externally unlabelled CO_2_ exchange through photosynthesis. At 15 DALV (Figs. 5a, 6a), each leaf pairs from lower two light intensities had the best amount of label enrichment (near to 100%) and equivalently the least amount of dilution. Indeed, unlabelled carbon fixation constantly enhanced with increasing light intensity, increasing leaf age and into the newer leaves. As a consequence, higher (35–45%) label dilutions were observed in the 3rd leaf pair at 20 DALV under higher light intensities (20 and 30 µmol m^−2^ s^−1^) (Fig. 6b).

Real-time measurement of carbon dioxide assimilation rate in 15 days old leaves from these cultures were also assessed for better understanding of the isotopic dilution due to the fixation of ambient carbon dioxide (Additional file 1: Table S3). Peppermint plants consumed less carbon dioxide under reduced light conditions (5 and 10 µmol m^−2^ s^−1^). However, carbon dioxide assimilation was relatively higher (0.433 and 0.518 µmol_CO2_ m_leafarea_^−2^ s^−1^) at high light intensities (20 and 30 µmol m^−2^ s^−1^, respectively).

### Establishing the effects of different light intensities on glucose consumption

**Fig. 7:**
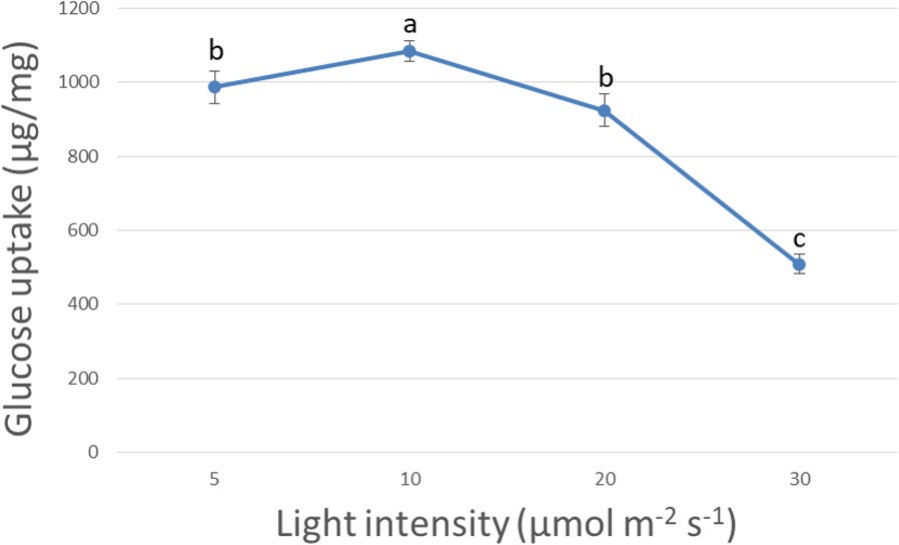
Comparison of glucose uptake (μg/mg leaf fresh weight) in shoot tip culture. Glucose uptake amount was measured at 15 DALV growth of the shoot culture under different light intensity. For statistics, data were evaluated by one-way Anova, followed by Tukey HSD test and statistical difference (p < 0.05) was indicated by different letters (a to c) (mean ± standard error, n = 5)

**Fig. 8:**
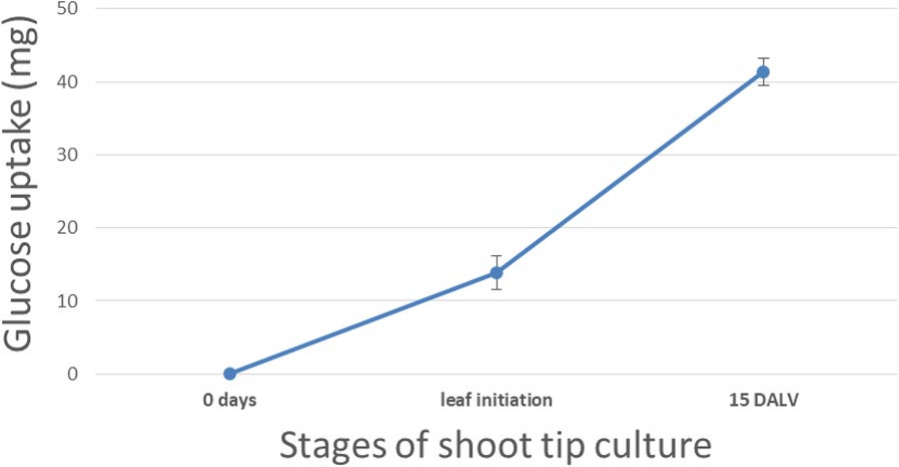
Biosynthetic need (mg) of glucose per shoot tip culture during different growth stages. Glucose uptake was measured during different growth stages up to 15 DALV at 5 μmol m^−2^ s^−1^ light intensity (mean ± standard error, n = 5)

The glucose uptake was determined in these shoot tip culture experiments, in order to understand the biosynthetic demand of carbon from sugar in the basal media rather than environmental CO_2_ fixation. After 15 DALV, 987 and 1084 µg glucose was consumed by the cultured explants under 5 and 10 µmol m^−2^ s^−1^ light, respectively to produce one mg leaf. However, glucose uptake amounts were progressively reduced at 20 µmol m^−2^ s^−1^ light intensity followed by further reduction at the highest light condition (Fig. 7). Analysing the glucose demands at 5 µmol m^−2^ s^−1^ light intensity across different developmental stages of the shoot indicated that 14 mg of glucose was consumed by each explant up to leaf initiation stage. Afterwards, uptake rate was increased and 41 mg was consumed up to 15 DALV (Fig. 8).

### Validation of the method

**Fig. 9:**
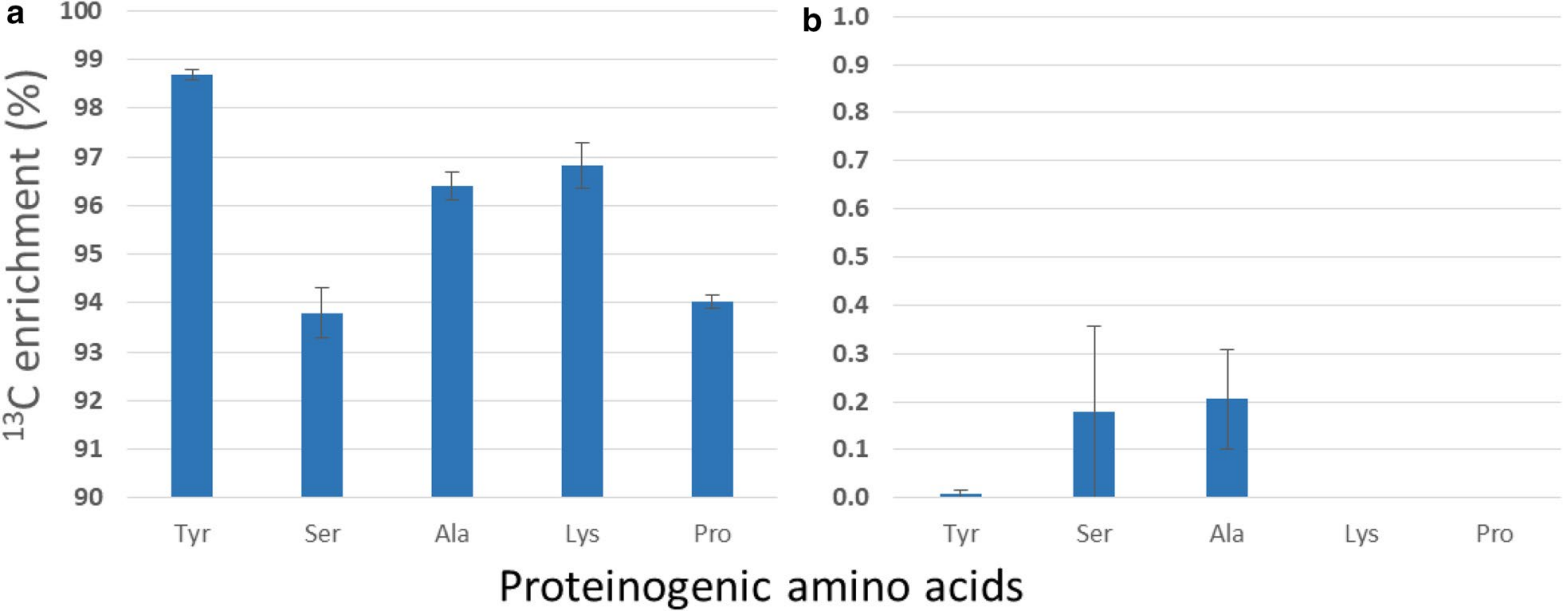
Average label enrichment (%) into different protein-bound amino acids in leaves of 15 DALV. **a** Cultured with U-13C glucose **b** Cultured with 12C glucose (control) at 5 μmol m^−2^ s^−1^ light intensity (mean ± standard error, n = 5)

Throughout the growth and development of a biological system, amino acids are produced from different parts of the central carbon metabolism such as glycolysis, pentose phosphate pathway and tricarboxylic acid cycle (Additional file 1: Table S4) [30]. These amino acids are translated into proteins. Thus, label incorporation throughout the whole system could be validated by the isotope inclusion into such proteinogenic amino acids. ^13^C enrichment in five protein-bound amino acids was evaluated using uniformly labelled glucose in the medium under best labelling condition (Fig. 9). Enrichment of labelled carbon was robust, varying from 94 to 99%. This validated the result of monoterpenes and confirmed that mint shoot tip culture under low light intensity was predominantly dependant on the supplied glucose. The sources of unlabelled carbon in amino acids were assumed to be due to 1% ^12^C impurities of labelled glucose, photosynthetically fixed atmospheric carbon or translocation of unlabelled amino acids from the initial explant shoot to the new sink leaves.

The shoot-tip culture strategy was also further validated in oregano plant under 10 µmol m^−2^ s^−1^ light intensity. These explants showed no visible leaf emergence at lower light intensities. Dilution of ^13^C isotope into monoterpene was found to be limited (1.36–1.83%) in growing shoot tips at 15 DALV (Additional file 1: Table S5).

## Discussion

### A metabolic sink system with enriched carbon isotope content

The main concept of sink and source leaves is that sinks are net importers, while the latter ones are net exporters of carbon assimilates. There are two kinds of the sink, namely utilization sinks (meristem, immature leaves) and storage sinks (tuber, embryo, seed). The utilization sinks are metabolically active and can also be referred to as metabolic sinks. When fed with isotopic tracers, these metabolic sinks provide a unique system to study changes in plant metabolic networks. Firstly, when one or more source leaves existed in the short-term ^13^C tracer study using the shoot tip culture system, a larger dilution effect from the unlabelled biomass of the already existing leaves towards ^13^C enrichment was observed (Figs. 1a, 2a). The reason for such a negative effect in the new sink leaves was mainly due to the additional import of unlabelled carbon from the existing source leaves. In the latter strategy without any source leaf, the newly developed immature leaves showed high label enrichment as there were no initial source leaves, except for 0.5 cm of shoot explant, which otherwise could have led to label dilution. Therefore, the biomass of the first leaf pair was nearly exclusively built from the externally supplied, labelled glucose. This technique of initiation of a culture without any existing (source) leaves exhibited more than threefold higher label enrichment in monoterpenes, compared to the system with previously existing leaves (Fig. 2).

### A system for further improvement towards ^13^C-MFA

In a growing plant, photosynthetic activity of the leaf increases with its age [31]. Through our system, we proved that modulating the intensity of light had a respective effect on external CO_2_ fixation. In this study, leaves grown under the highest experimental light intensity indirectly exhibited ambient CO_2_ consumption, even at half leaf expansion stage (15 DALV). Previous observations demonstrated that peppermint leaves expanded to half size after 15 days and to full size after 21 days [32]. Thereupon, isotopic dilution due to unlabelled CO_2_ fixation was varied from 17 to 38% (Fig. 6a). This result was supported by the previous reports about the transition from predominantly sink leaves to predominantly source leaves after 30–60% of the leaf age [32, 33]. Within this transition, leaves change from net carbon importers to net carbon exporters. However, label dilution due to fixation of unlabelled CO_2_ was very limited under the lower two light conditions at half leaf expansion stage (Fig. 6a). It can be concluded that the first pair of leaves at these two reduced light conditions acted as metabolic sinks due to a limitation of light reactions. The amount of sucrose available in source leaves for transporting to sink tissues depends on photosynthetic activity [34], which is quite identical at higher light (30 µmol m^−2^ s^−1^) intensity. This is in contrast to reduced light conditions (5 or 10 µmol m^−2^ s^−1^). Transported sugars from pre-mentioned sources (first leaf pair after 15 DALV) to new sinks (second leaf pair after 15 DALV) was mostly made from external ^13^C glucose under low light. The glucose consumption data also indicated that at a higher light intensity, our shoot tip explants employed photosynthesis to satisfy their carbon demands partly, whereas at lower light intensities they showed increased dependence on the externally supplied glucose (Fig. 7). Previous studies confirmed that upregulation of hexose content causes downregulation of photosynthesis [35–38]. This was further corroborated with real time measurement of photosynthetic rate via carbon dioxide assimilation studies on 15 days old leaves (Additional file 1: Table S3). Peppermint plants showed negligible carbon dioxide uptake rates under reduced light conditions (5 and 10 µmol m^−2^ s^−1^). However, at a higher light intensity (20 and 30 µmol m^−2^ s^−1^) they showed increased carbon dioxide assimilation. Thus, our low light shoot tip system can avoid photosynthetic label scrambling for ^13^C MFA in plants.

Our system also displayed competent growth as new leaves emerged, although at a slower rate, however with similar volatile and pigment content, compared to the plants grown at ambient light conditions (Additional file 1: Table S2). Despite the fact that low light ensured a reduction of photosynthesis over 2 weeks, the amount of various phytopigments remained very similar under different light intensities. Besides, statistically similar chlorophyll a/b ratio at normal and 5 µmol m^−2^ s^−1^ light was indicating that the peppermint plants could adapt to the low light. In addition, no remarkable differences were observed in the terpenoid content. This suggested that our shoot tip culture system was very stable over time and that altering the light intensity did not negatively affect the growth capacity of the explant while simultaneously resulting in a high label enrichment.

The high label in hydrolysed amino acids further proved the efficacy of our shoot tip system to be used as an in vitro system for ^13^C MFA. Label in different amino acids were varied up to only 5%. It might be possible that some amount of unlabelled amino acids were transported from the explant shoot tip to the sink tissue for the initial growth of shoot culture [13, 39]. Hence, the amount of e.g. ^13^C in serine and lysine are slightly different from the label in tyrosine. Amino acids are produced by different metabolic pathways and in different compartments of the cell. As an instance, the label amount in tyrosine, which is produced in the plastid [30], strongly represents the effect of photosynthesis. More than 98.5% label in tyrosine suggested that this culture system relied on external glucose over 15 days of leaf growth, rather than CO_2_ fixation (Fig. 9). Tyrosine, serine, alanine, lysine and proline are also synthesised from different intermediates of several primary metabolic pathways as described in Additional file 1: Table S4. Besides, these amino acids are produced in different cellular compartments such as cytosol, plastids and mitochondria. A very high percentage of isotopic inclusion in proteinogenic amino acids was also noticed (Fig. 9). Hereby, this study illustrated high ^13^C enrichment throughout the plant metabolic network and various subcellular compartments. Eminent amount of label in these amino acids highly recommend the use of our system in carbon-based metabolic flux analysis to study the metabolism of developing leaves.

Plants have a complex metabolic system where the matrix effect of interaction between various inter- or intra-compartment metabolic pathways at cell level are apparent [40]. Indeed, investigation of concerned metabolic pathways in the normal and continuously growing plant is much more realistic than in the specific organ culture. Secondary metabolite production requires a longer time period. Maintaining high ^13^C enrichment and isotopic steady state over longer time periods are challenging for steady state MFA, due to impoverished labelling amounts on exposure to light. Therefore, on previous occasions, time-course labelling experiments were undertaken for instationary MFA study on plant secondary metabolism [20]. Here in this study, our shoot tip culture system enabled us to maintain the growing plants for over 2 weeks with virtually 100% isotopic enrichment in monoterpenes, thereby extending the area in steady-state flux analysis of plant secondary metabolism. Interestingly, this system can also be implemented in other plants, however, it may warrant other necessary alterations in light intensities or growth hormone addition. As an instance, oregano plant grown under our shoot tip culture system with 10 µmol m^−2^ s^−1^ light intensity showed very low isotopic dilution (Additional file 1: Table S5).

### A system to study sink-source transition in leaves

Young leaves are mixotrophic, growing partially depending on the carbohydrates imported from other organs of the same plant. Mature leaves, on the other hand, are autotrophic, producing an excess of photo-accumulates and acting as the plant’s major sources for transportable sugar [35]. As the young leaves develop into mature leaves, the effect of photosynthesis increases and carbohydrate metabolism is switched from catabolic to anabolic pathways. During ontogeny, leaves undergo a developmental transition from net importers (sink) to net exporters (source) of photoassimilates [41]. In this transition, minimum three different phases are observed. At an early immature phase, the leaves are fully dependent on the source for their carbon need and act predominantly as sink tissue. After some days, at a late immature phase, the leaves are capable to make their own carbon with increasing photosynthetic activity, although they are still mostly dependent on other source leaves and remain as a sink. Finally, immature leaves become mature and transform themselves to become the source tissue for new sink leaves due to their own photosynthetic assimilation [35]. In our system, we observed that the leaves after 15 DALV at 5 and 10 µmol m^−2^ s^−1^ light (Fig. 5a) behaved like the first phase of sink tissue where they were almost fully dependent on the external isotopic carbon source. The best portrait of photosynthetic activity in the middle phase of sink-source transition could be demonstrated in leaves of 20 DALV age at the lowest light intensity (Fig. 5b) when the cultured plant started to make photoaccumulates and therefore isotopic enrichment was decreased, nevertheless labelled glucose was still the major source for carbon supplies. We also observed the effect of photosynthesis on the final phase when ^13^C enrichment of monoterpene in the third leaf pair at 30 µmol m^−2^ s^−1^ light intensity was reduced to 54% (Fig. 5). A plausible idea is suggested here that the first pair of leaves, which had started to photosynthesize, acted as source leaves by transporting the sugars to the 3rd pair of leaves and thereby diluting the labelled carbon. This finding indicated that the U-^13^C_6_ glucose study with altered light intensity allowed us to gain a deeper understanding of the sink to source transition of developing mint leaves. Our system, therefore, provides an excellent tool for investigating sink leaf metabolism [42, 43].

## Conclusions

In summary, we established a system where increased amounts of volatile terpenes and high sink strength were maintained under the limited light condition even after 2 weeks; thus indicating a positive outlook of our methodology. With this study we provide a concrete step forward towards accurate ^13^C MFA for analyzing complex plant metabolism in growing plant system. It can be explored further to probe various primary as well as secondary metabolic pathways as well as to study metabolism across different time durations. Using this culture system, performing ^13^C MFA can be less challenging and more effective to quantify the contribution of different pathways for various plant products. In addition, the carbon economy of the plant can also be conceived. Primary metabolism in leaves does not demand long-term experiments, and in our study, the ^13^C incorporation level in proteinogenic amino acids was exceptionally high already after 2 weeks. Therefore, this system also provides an alternative approach for studying steady-state fluxes in primary metabolism for shorter time-periods. Our culture system is economically feasible (Additional file 2), robust and allows parallel operation without any requirement of specialized equipment during cultivation.

## Methods

### Chemicals and plant materials

The labelling studies were performed using U-^13^C_6_ glucose (99% purity, Campro Scientific GmbH, Germany). Hexokinase (HK), glucose-6-phosphate dehydrogenase (G6PDH), nicotinamide adenine dinucleotide (NAD) and adenosine triphosphate (ATP) which were required for the enzymatic assays for glucose estimation were obtained from Sigma-Aldrich, USA. The required plant materials, peppermint (*Mentha* × *piperita* var. Multimentha) and oregano (*Origanum vulgare*) were obtained from Dehner garden center (Halle, Germany). They were maintained in the growth chambers (Microclima MC1000, Snijders Lab, Netherlands) at 25 ± 1 °C, 65% relative humidity and 400 µmol m^−2^ s^−1^ light with 16 h photoperiod.

### Shoot tip culture

The shoot tip culture of peppermint was performed with some modifications from the protocol described by [44]. Shoot apices were taken from 5 to 6 weeks grown healthy plant to maintain the same vegetative growth and avoid the reproductive stage of the plant. Shoot apices with about 1 cm from the adjacent stem were cleaved from the plant and sterilized with 70% ethanol for 60 s. Subsequently, these apices were treated with 0.5% sodium hypochlorite solution, containing 100 µl/l Tween 20, for 7 min. This was followed by 4 times repeated rinsing with autoclaved distilled water. Then the end of the stem which was blackened (dead) due to its exposure to sterilization and washing solutions, was trimmed away. The final apex (about 5-7 mm) with none and/or one developing leaf pair was used as an explant which was then cultured inside the Magenta box (Sigma-Aldrich, Germany), containing the MS basal media [45], supplemented with B5 vitamins. Fully labelled (U-^13^C_6_) or unlabelled (^12^C) glucose was added to the basal MS medium for qualitative or quantitative analysis, respectively. These cultures were cultivated in a growth chamber (CU-22L, Percival Scientific Inc, USA) at 25 ± 1 °C and a 16/8 h light/dark photoperiod. Irradiation with different light intensities (5, 10, 20 or 30 µmol m^−2^ s^−1^) was provided by Osram cool white fluorescence lamps (Lumilux T8 L18 W/840, Germany). Samples were collected on the 15th or 20th day after first leaf visibility (DALV). Plant leaf samples (~ 40–50 mg) were flash frozen in liquid nitrogen and stored at − 80 °C for further investigation. For label enrichment analysis, different pairs of leaves were collected separately; however, total leaves were stored collectively for other studies. Time for initiation of the culture (14.00–15.00 h CEST) and sample collection (10.00–11.00 h CEST) was fixed to get the constant influence of diurnal rhythm for every treatment, which is known to have a role for the non-mevalonate pathway [46, 47].

The oregano shoot-tip culture was performed with the same strategy under 10 µmol m^−2^ s^−1^ light intensity, as this seemed to be the lowest possible light intensity for adequate growth of the explant.

### Measurement of terpenoids

Before analysis, frozen leaf samples were grounded in liquid nitrogen with the help of micro pestles within microcentrifuge tubes. For qualitative analysis of terpenoids, the homogenized freeze-dried sample was collected into a 1.5 ml SPME (Solid Phase Micro Extraction) vial. A 100 µm polydimethylsiloxane SPME fiber (Supelco Analytical, Sigma-Aldrich, USA) was exposed to the headspace of the sample for 2 min at ambient temperature. For quantitative analysis of the terpenoids, fresh weight of the sample was determined prior to it being homogenized. Further 300 µl of hexane containing 10 ng/l of nonyl acetate (as an internal standard) was added to the vial. The mixture was shaken overnight at 25 °C. The following morning, the mixture was then allowed to stand at room temperature for 10 min before collecting the supernatant for further quantitative analysis.

Samples were analyzed with a GCMS 2010 gas chromatograph, coupled to GCMS-QP 2010 Plus mass spectrometer (Shimadzu Corporation, Japan). For the quantitative analysis, a split injection was used with a ratio of 1:5, whereas for qualitative analysis the unloading of the SPME fibers was carried out in splitless mode. Analytic separation was performed in Supreme-5 ms column (30 m × 0.25 mm × 0.25 µm) (Chromatographie Service GmbH, Germany). Hydrogen was used as a carrier gas with a flow rate of 1 mL/min. Injector and interface temperature were respectively set to 220 °C and 250 °C with an ionization potential of 70 eV and a scan range of 50–350 amu. The oven temperature was held at 50 °C for 3 min, then increased with 7 °C/min to 150 °C followed by a rate of 100 °C/min up to 300 °C, which was held for 2 min. The sampling and solvent cut time were adjusted to 1 min and 1.5 min, respectively.

### Labelled data interpretation procedure

The GCMS Postrun Analysis software was used to analyze the chromatograph and the Adam’s library (Adams, 2007) was integrated into the software for the identification of peaks [48]. Terpenoids were identified from control unlabelled sample by mass spectral libraries and those from the labelled sample were distinguished by comparing retention time with the control mass spectra peaks. In case of qualitative interpretation, the intensity of each mass isotopomer, i.e. (M + 0) to (M + 10) isotopomers for each monoterpene (C_10_ compound) was corrected for theoretical natural abundance. Corrected Abundance (C) of each mass isotopomer was normalized to mass isotopomer distribution (MID) which was the percentage of corrected abundance for each isotopomer as a fraction of the total abundances for all isotopomers. MID can be defined by Eq. (1), and Eq. (2) was the instance for MID of (M + 0) isotopomer.

1 $$ MID \left( D \right) = \frac{C}{{\mathop \sum \nolimits_{i = 0}^{n} \left( {M + i} \right)}} $$

2 $$ MID_{{\left( {M + 0} \right)}} = \frac{{C_{{\left( {M + 0} \right)}} }}{{\mathop \sum \nolimits_{i = 0}^{n} \left( {M + i} \right)}} $$

The number of carbon atoms present in the compound was symbolized by n. Average ^13^C enrichment was enumerated by summing up each MID after multiplication with their extra ^13^C atom number as the fraction of a total atom as follows in Eq. (3).

3 $$ Average\,13C\,Enrichment\,\left( E \right) = \frac{{\left\{ {MID_{{\left( {M + 0} \right)}} \times 0} \right\} + \left\{ {MID_{{\left( {M + 1} \right)}} \times 1} \right\} + \cdots + \left\{ {MID_{{\left( {M + n} \right)}} \times n} \right\}}}{n} $$

To determine scrambling of the label due to external carbon dioxide exchange and presence of unlabelled carbon in initial biomass of 5–7 mm stem, ^13^C enrichment was recalculated after correcting not only natural abundance but also 1% U-^13^C_6_ glucose impurity. After these two corrections, average dilution of labelling (F) was measured by Eq. (4)

4 $$ Dilution\,of\,labelling\,\left( F \right) = 100\% - Corrected\,13C\,Enrichment $$

### Measurement of photosynthetic pigment contents

Chlorophyll and carotenoid content were measured as described by [49]. Quenching leaves were weighted and fresh weight (FW) was recorded. Weighted samples were further homogenized using micro pestles. Two ml of 95% (v/v) ethanol was added to the pulverized tissue and samples were vortexed for 30 s. These tubes were then left overnight at room temperature after wrapping with aluminium foil to prevent photobleaching of the analyzed pigments. Samples were vortexed and left standing for 10 min, prior to the measurement. The final analysis was performed with 1 ml clear supernatant. The light spectroscopy (GeneQuant™ 1300 spectrophotometer, Healthcare Bio-Science AB, Sweden) was normalized with water and 95% ethanol as blank. The supernatant was measured at 470 (A_470_), 649 (A_649_) and 664 (A_664_) nm absorbance to get the quantitative measurement of chlorophylls and total carotenoids. Additionally, the sample was measured at 750 nm to correct the measurement error. The content of individual pigments was calculated with the following formula [49],

5 $$ Cholorophyll a \,\left( {C_{a} } \right) = \frac{{\left( {13.36 A_{664} - 5.19 A_{649} } \right) \times 2}}{FW} $$

6 $$ Cholorophyll b \,\left( {C_{b} } \right) = \frac{{\left( {27.43 A_{649} - 8.12 A_{664} } \right) \times 2}}{FW} $$

7 $$ Carotenoids \,\left( {C_{x + c} } \right) = \frac{{\left( {4.785 A_{470} + 3.657 A_{664} - 12.76 A_{649} } \right) \times 2}}{FW} $$

### Measurement of glucose uptake

After 15 DALV, remaining medium from each shoot tip culture set up was measured and stored at -20 °C. Glucose uptake was assayed using the modified version of the original protocol [50, 51]. A buffer containing 100 mM imidazole–HCl (pH 6.9), 5 mM MgCl_2_, 2.25 mM NAD, 1 mM ATP as final concentrations were used for the measurement of soluble sugars using EL×808 ultramicroplate reader (BioTeK Inc, Germany) at 340 nm. Temperature of the plate reader was set at 30 °C. Further addition of auxiliary enzymes such as glucose 6-phosphate dehydrogenase and hexokinase, allowed the removal of endogenous hexose-phosphates and measurement of glucose respectively. A calibration curve with different concentrations of glucose was used to measure the glucose amounts in leftover MS medium. Glucose uptake was further determined by deducting the leftover glucose from the total supplemented glucose.

### Measurement of isotope enrichment in protein hydrolysates

The method was validated by studying the isotope enrichment in proteinogenic amino acids of the total leaf pairs, which were cultivated in U-^13^C_6_ glucose media under 5 µmol m^−2^ s^−1^ light intensity for 15 DALV. Proteins were hydrolyzed in 6 N HCl for 16 h at 100 °C as described previously [52]. These amino acids were derivatized to N,O-*tert*-butyldimethylsilyl (TBDMS) derivatives. Amino acids in these storage proteins were analysed by GCMS (7890B GC, 7200 QTOF, Agilent Technologies Inc, USA). Sample was splitlessly injected in 250 °C heated liner. Zebron Capillary GC-Column (ZB-Semi Volatiles, 30 m × 0.25 mm × 0.25 μm, Phenomenex, Torrance, USA) was used for analytical separation. Helium carrier gas was provided at a constant flux of 1 ml/min. The oven temperature was initially held at 60 °C for 2 min, then increased to 280 °C at 20 °C/min rate and finally held for another 5 min. Mass spectrometer had 230 °C heated source with an ionization potential of 70 eV. Amino acids were detected at scan mode in the range of 50-550 amu and rate was at 5 spectra/s. List of measured fragments was presented in Additional file 1: Table S4. Data were evaluated by Mass Hunter Qualitative Analysis software (B.07.00, Agilent Technologies Inc, USA).

## Additional files

**Additional file 1: Figure S1.** Mass isotopomer distribution (%) of isopulegone in each leaf pair at 30 μmol m^-2^ s^-1^ light intensity. (a) Shoot-tip culture was initiated with one developing leaf pair and after 15 days of culture, total four leaf pairs (older than 6 days) were observed. (b) Shoot-tip culture started without leaves and after 15 DALV, total three leaf pairs (older than 6 days) were observed (mean ± standard error, n = 5). **Figure S2**. Mass isotopomer distribution (%) of menthofuran in each leaf pair at 30 μmol m^-2^ s^-1^ light intensity. (a) Shoot-tip culture initiated with one developing leaf pair and after 15 days of culture, total four leaf pairs (older than 6 days) were observed. (b) Shoot-tip culture started without leaves and after 15 DALV, total three leaf pairs (older than 6 days) were observed (mean ± standard error, n = 5). **Figure S3**. Photographs of shoot-tip culture (a) Shoot-tip culture at 15 DALV under 5 μmol m^-2^ s^-1^ light intensity. (b) Shoot-tip culture at 20 DALV under 5 μmol m^-2^ s^-1^ light intensity (c) Overview of shoot- tip culture system inside growth chamber. Two and three leaf pairs (older than 6 days) were observed at 15 and 20 DALV, respectively. **Figure S4**. Mass isotopomer distribution (%) of pulegone in control shoot-tips, nurtured with unlabelled glucose as sole carbon source. Calculated label dilution in this control shoot-tips was 99.74% (after natural abundance correction) with negligible amounts of ^13^C (0.26%) was considered as an experimental or measurement error (mean ± standard error, n = 5). This result remained virtually similar in control treatments within leaves of different age grown under different light intensities. **Table S1**. Consumption of glucose (mg) by shoot-tip culture. Glucose consumption was measured after 15 DALV growth of the shoot-tip culture under different light intensities (mean ± standard error, n = 5). **Table S2**. Production of photosynthetic pigments and volatile terpenes in 15 days old leaves from in vivo plants. Leaves were collected from the peppermint plant grown under natural condition (mean ± standard error, n = 5). **Table S3**. Photosynthetic assimilation rates of 15 days old leaves from shoot-tip cultures and normal plants. The measurements were taken using the LI-6400XT portable gas exchange system (Li-Cor Inc., Lincoln, NE, USA) (mean ± standard error, n = 5). **Table S4**. Details of measured amino acids and their fragments. **Table S5**. Label dilution (%) into monoterpene in two leaf pairs of oregano shoot-tip culture after 15 DALV under 10 μmol m^-2^ s^-1^ light intensity. Labelled U-13C glucose (2%) was supplemented in the basal media (mean ± standard error, n = 5).

**Additional file 2: Data S1.** Approximate cost calculation and time required for the shoot-tip culture method.

## Data Availability

All data generated or analysed during this study are included in this published article and its supplementary information files.

## Abbreviations

DALV: days after first leaf visibility
MFA: metabolic flux analysis
CEST: Central European Standard Time
