# Supplementary material for: Shoot tip culture: a step towards 13C metabolite flux analysis of sink leaf metabolism
Source: Plant Methods. 2019 May 20;15:48. doi: 10.1186/s13007-019-0434-8 (PMC6526604; doi:10.1186/s13007-019-0434-8)
Supplement: Supplementary file 1 — Additional file 1: Figure S1. Mass isotopomer distribution (%) of isopulegone in each leaf pair at 30 μmol m-2 s-1 light intensity. (a) Shoot-tip culture was initiated with one developing leaf pair and after 15 days of culture, total four leaf pairs (older than 6 days) were observed. (b) Shoot-tip culture started without leaves and after 15 DALV, total three leaf pairs (older than 6 days) were observed (mean ± standard error, n = 5). Figure S2. Mass isotopomer distribution (%) of menthofuran in each leaf pair at 30 μmol m-2 s-1 light intensity. (a) Shoot-tip culture initiated with one developing leaf pair and after 15 days of culture, total four leaf pairs (older than 6 days) were observed. (b) Shoot-tip culture started without leaves and after 15 DALV, total three leaf pairs (older than 6 days) were observed (mean ± standard error, n = 5). Figure S3. Photographs of shoot-tip culture (a) Shoot-tip culture at 15 DALV under 5 μmol m-2 s-1 light intensity. (b) Shoot-tip culture at 20 DALV under 5 μmol m-2 s-1 light intensity (c) Overview of shoot- tip culture system inside growth chamber. Two and three leaf pairs (older than 6 days) were observed at 15 and 20 DALV, respectively. Figure S4. Mass isotopomer distribution (%) of pulegone in control shoot-tips, nurtured with unlabelled glucose as sole carbon source. Calculated label dilution in this control shoot-tips was 99.74% (after natural abundance correction) with negligible amounts of 13C (0.26%) was considered as an experimental or measurement error (mean ± standard error, n = 5). This result remained virtually similar in control treatments within leaves of different age grown under different light intensities. Table S1. Consumption of glucose (mg) by shoot-tip culture. Glucose consumption was measured after 15 DALV growth of the shoot-tip culture under different light intensities (mean ± standard error, n = 5). Table S2. Production of photosynthetic pigments and volatile terpenes in 15 days old leaves from in vivo [file 13007_2019_434_MOESM1_ESM.pdf]

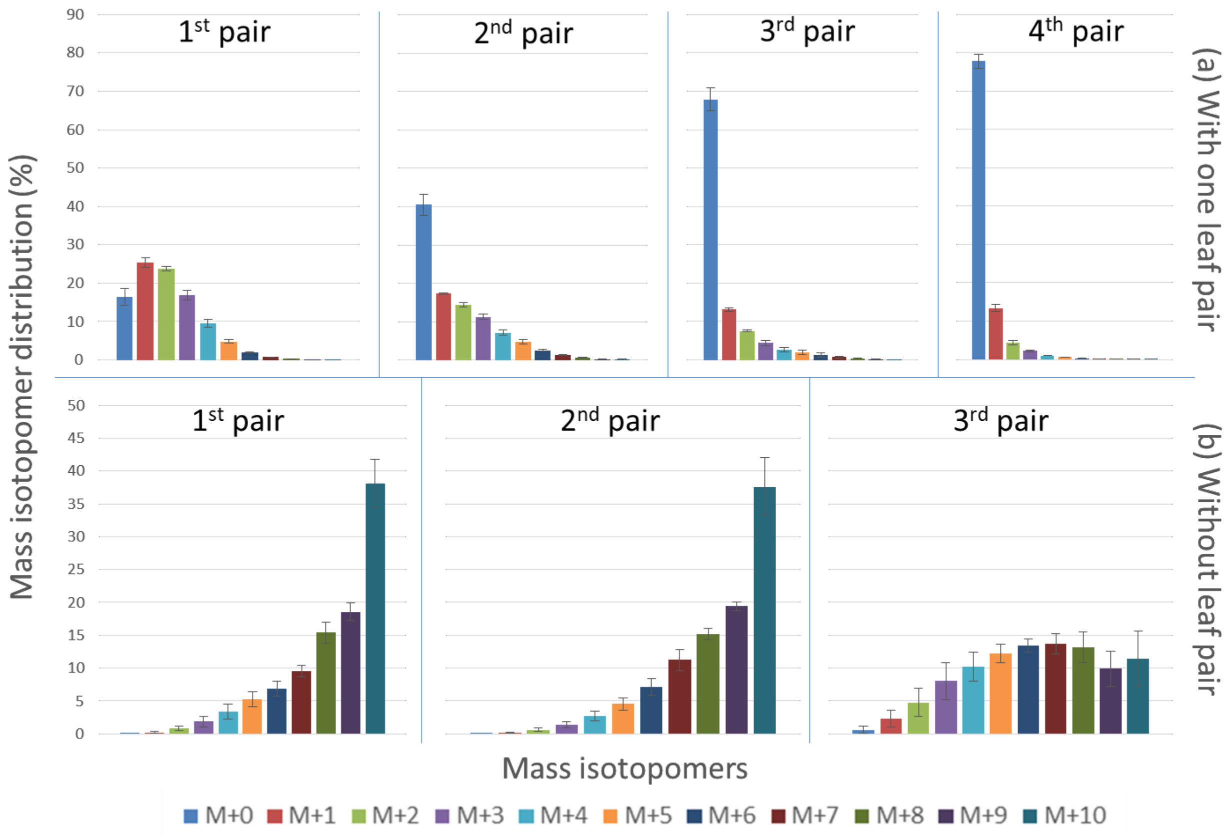

Figure S1. Mass isotopomer distribution (%) of isopulegone in each leaf pair at 30 µmol m^{-2} s^{-1} light intensity. (a) Shoot-tip culture was initiated with one developing leaf pair and after 15 days of culture, total four leaf pairs (older than 6 days) were observed. (b) Shoot-tip culture started without leaves and after 15 DALV, total three leaf pairs (older than 6 days) were observed (mean ± standard error, n=5).

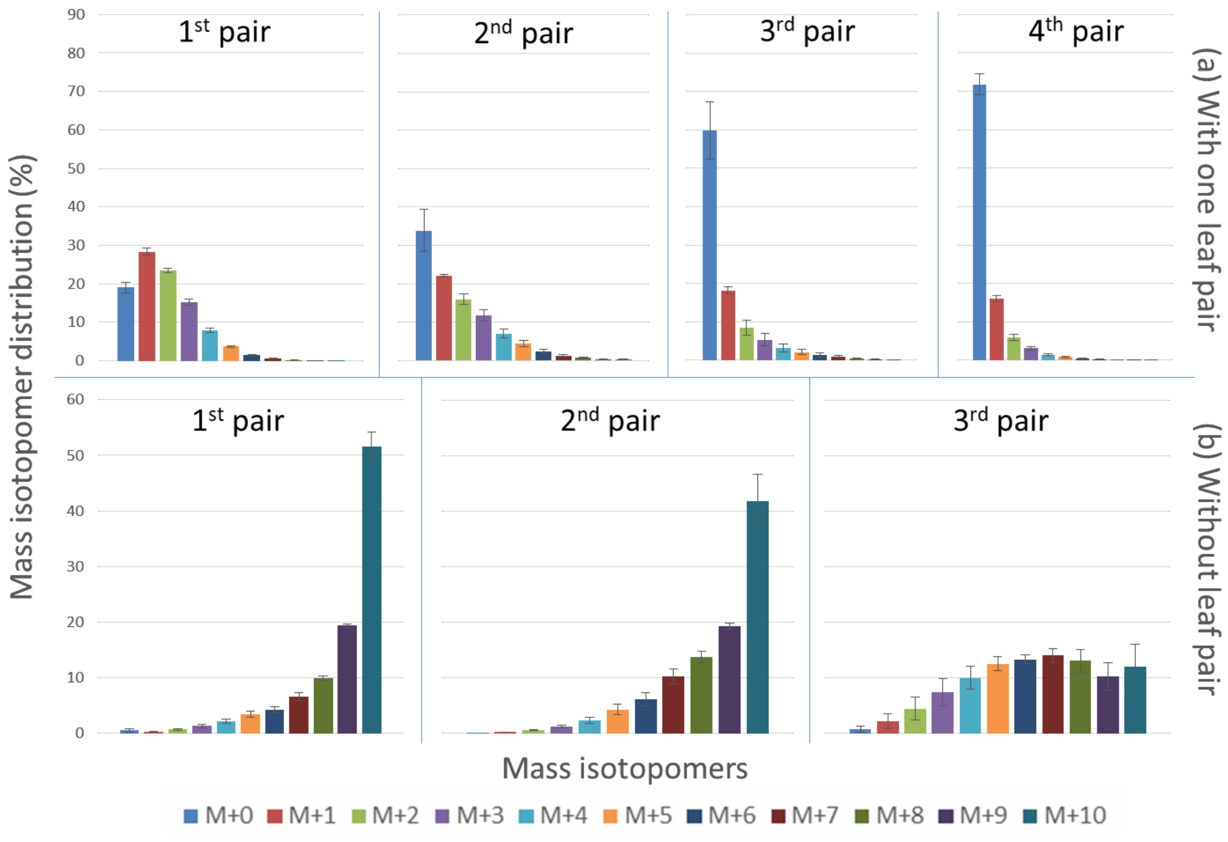

Figure S2. Mass isotopomer distribution (%) of menthofuran in each leaf pair at 30 µmol m^{-2} s^{-1} light intensity. (a) Shoot-tip culture initiated with one developing leaf pair and after 15 days of culture, total four leaf pairs (older than 6 days) were observed. (b) Shoot-tip culture started without leaves and after 15 DALV, total three leaf pairs (older than 6 days) were observed (mean ± standard error, n=5).

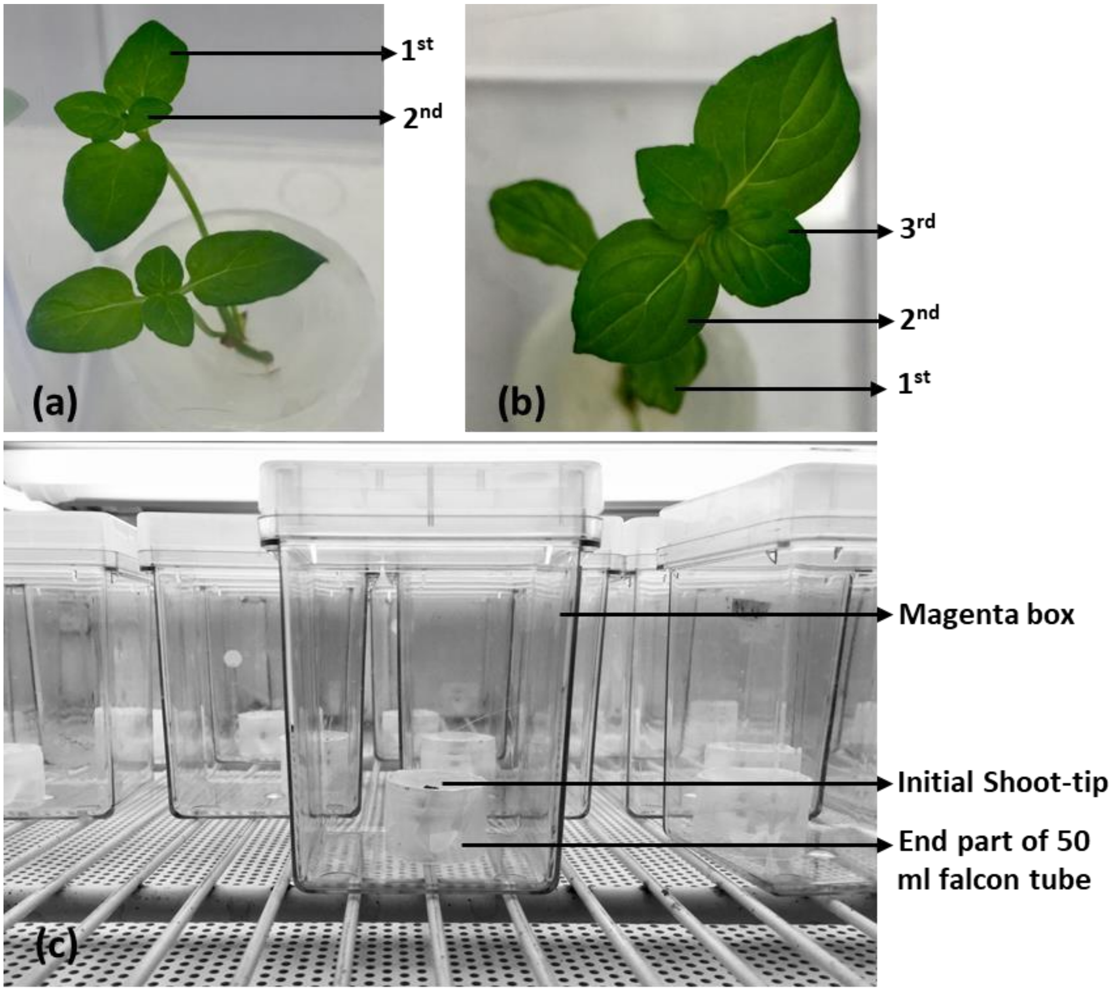

Figure S3. Photographs of shoot-tip culture (a) Shoot-tip culture at 15 DALV under 5 µmol m^{-2} s^{-1} light intensity (b) Shoot-tip culture at 20 DALV under 5 µmol m^{-2} s^{-1} light intensity (c) Overview of shoot- tip culture system inside growth chamber. Two and three leaf pairs (older than 6 days) were observed at 15 and 20 DALV, respectively.

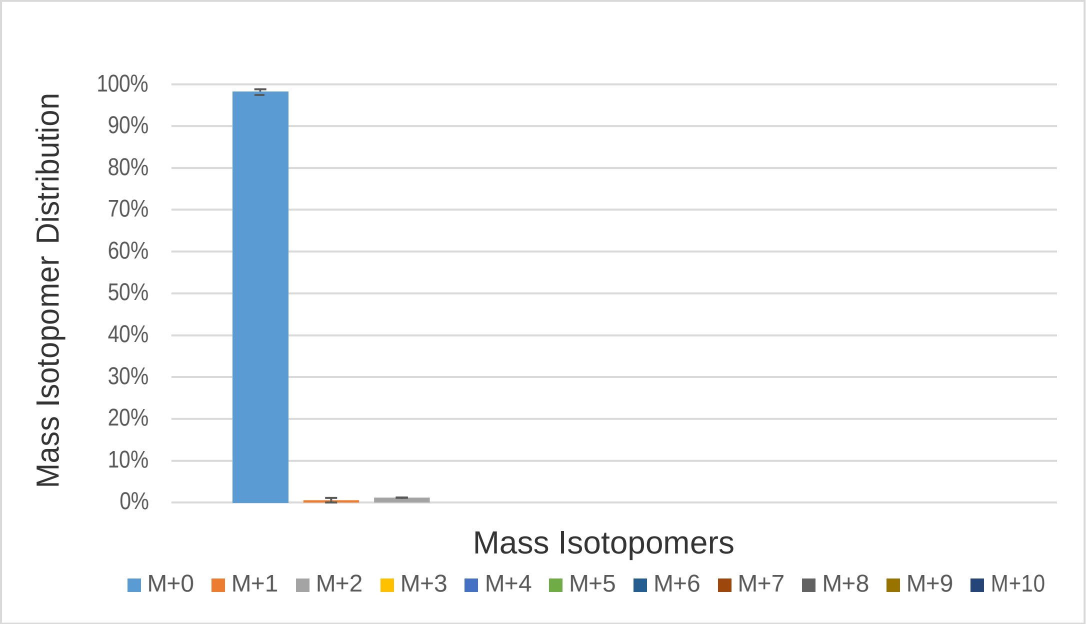

Figure S4. Mass isotopomer distribution (%) of pulegone in control shoot-tips, nurtured with unlabelled glucose as sole carbon source. Calculated label dilution in this control shoot-tips was 99.74% (after natural abundance correction) with negligible amounts of ^{13}C (0.26%) was considered as an experimental or measurement error (mean ± standard error, n=5). This result remained virtually similar in control treatments within leaves of different age grown under different light intensities.

Table S1. Consumption of glucose (mg) by shoot-tip culture. Glucose consumption was measured after 15 DALV growth of the shoot-tip culture under different light intensities (mean ± standard error, n=5).

**Light intensity (μmol m^{-2} s^{-1})**	**Total glucose needed (mg/shoot culture)**
5	41.4±1.8
10	47.1±1.2
20	54.8±2.6
30	46.3±2.4

Table S2. Production of photosynthetic pigments and volatile terpenes in 15 days old leaves from *in vivo* plants. Leaves were collected from the peppermint plant grown under natural condition (mean ± standard error, n=5).

**Metabolites**	**Quantity**
Chlorophyll (mg g^{-1} leaf fresh weight)	
Chl a	1.53 ± 0.03
Chl b	1.00 ± 0.07
Total (a+b)	2.54 ± 0.09
Chl (a/b)	1.54
Carotenoids (mg g^{-1} leaf fresh weight)	0.31 ± 0.02
Volatile terpenes (ng mg^{-1} leaf fresh weight)	
Pinene α	163.38 ± 27.92
Sabinene	78.90 ± 13.28
Pinene β	315.86 ± 49.07
Myrcene	90.27 ± 16.37
Limonene	33.70 ± 6.83
1,8-Cineole	1110.72 ± 161.46
Sabinene Hydrate	271.72 ± 59.57
Menthone	9599.41 ± 2651.2
Menthofuran	2226.18 ± 346.1
Isopulegone	684.64 ± 65.57
Pulegone	2846.56 ± 262.95
Germacrene D	91.89 ± 15.84
Total volatile terpene	17513.25 ± 3333.95

Table S3. Photosynthetic assimilation rates of 15 days old leaves from shoot-tip cultures and normal plants. The measurements were taken using the LI-6400XT portable gas exchange system (Li-Cor Inc., Lincoln, NE, USA) (mean ± standard error, n=5).

**Light intensity (μmol m^{-2} s^{-1})**	**Assimilation rate (μmol $_{(CO2)}$ m^{-2} $_{(leaf\ area)}$ s^{-1})**	
	Shoot-tip cultures	Normal plants
5	-0.402 ± 0.094	-0.482 ± 0.114
10	-0.129 ± 0.089	-0.101 ± 0.056
20	0.433 ± 0.082	0.461 ± 0.123
30	0.518 ± 0.077	0.661 ± 0.112

Table S4. Details of measured amino acids and their fragments

**Measured amino acids**	**Source intermediate(s)**	**Compartments** (Dersch et al., 2016)	**Analysed fragments**
Tyrosine (Tyr)	Phosphoenol pyruvate and Erythrose 4-phosphate	Plastid	364 (M-57)
Serine (Ser)	3-phosphoglyceric acid	Cytosol	362 (M-85)
Alanine (Ala)	Pyruvate	Cytosol	232 (M-85)
Lysine (Lys)	Oxaloacetic acid	Cytosol	431 (M-57)
Proline (Pro)	α-ketoglutarate	Mitochondria	258 (M-85)

Table S5. Label dilution (%) into monoterpene in two leaf pairs of oregano shoot-tip culture after 15 DALV under 10 µmol m^{-2} s^{-1} light intensity. Labelled U-^{13}C glucose (2%) was supplemented in the basal media (mean ± standard error, n=5).

**Leaf pair**	**Label dilution (%)**
First	1.83 ± 0.19
Second	1.36 ± 0.05
