# Supplementary material for: Shoot tip culture: a step towards 13C metabolite flux analysis of sink leaf metabolism
Source: Plant Methods. 2019 May 20;15:48. doi: 10.1186/s13007-019-0434-8 (PMC6526604; doi:10.1186/s13007-019-0434-8)
Supplement: Supplementary file 2 — Additional file 2: Data S1. Approximate cost calculation and time required for the shoot-tip culture method. [file 13007_2019_434_MOESM2_ESM.pdf]

Data S1. Approximate cost calculation and time required for the shoot-tip culture method

A. One-time cost of reusable consumables for experiment with 5 replications:

Magenta boxes- 11 EUR

Cut end of falcon tubes - 1.5 EUR

B. Cost of non-reusable consumables for experiment with 5 replications:

½ conc. MS buffer – ~5-10 cents

U-^{13}C glucose - ~ 40 EUR

Parafilm- ~10-15 cents

C. Time required for the shoot-tip set-up:

Autoclaving of the magenta boxes & clean bench UV sterilization: 1-2 hours

Shoot-tip isolation - ~2 mins

Shoot-tip sterilization - 12 mins

Shoot-tip system set-up - ~5 mins
